# Respiratory syncytial virus prophylaxis for children in Africa: Challenges, opportunities and public health strategies

**DOI:** 10.4102/jphia.v16i1.1251

**Published:** 2025-05-23

**Authors:** Phillip T. Chigiya

**Affiliations:** 1 Department of Public Health, Dawa Health Clinic, Lusaka, Zambia

**Keywords:** respiratory syncytial virus, maternal vaccination, monoclonal antibodies, vaccine hesitancy, Africa, surveillance, public health

## Abstract

Respiratory syncytial virus (RSV) is a leading cause of severe lower respiratory tract infections (LRTIs) in young children, accounting for an estimated 94 600 to 149 400 deaths annually and over 33 million cases of LRTI. The burden is particularly acute in Africa, where limited healthcare access, malnutrition, and co-infections exacerbate outcomes. Despite the introduction of maternal vaccines, such as RSVpreF (respiratory syncytial virus prefusion F protein vaccine), and monoclonal antibodies (mAbs), such as nirsevimab, barriers including high costs, infrastructure limitations, and vaccine hesitancy hinder implementation in African settings. This article examines the challenges of RSV prophylaxis in Africa, including the economic burden of interventions, cold chain requirements, and the scarcity of robust epidemiological and surveillance data. It highlights the need for expanded molecular surveillance and localised clinical trials to ensure the safety and efficacy of these interventions. Vaccine hesitancy, rooted in historical failures such as the formalin-inactivated RSV vaccine, underscores the importance of culturally sensitive community engagement. Opportunities for advancing RSV prevention in Africa include integrating maternal vaccines into antenatal care systems, aligning vaccination schedules with RSV seasonality, and leveraging private sector partnerships. Advocacy for WHO prequalification is essential to enable global procurement and secure international funding. A dual approach combining maternal vaccines with mAbs offers comprehensive protection, particularly for high-risk infants. By addressing these challenges and leveraging available opportunities, Africa can lead efforts to reduce RSV-associated morbidity and mortality, improving outcomes for its most vulnerable populations.

## Introduction

Respiratory syncytial virus (RSV) represents a significant public health challenge, particularly for infants under 6 months of age who experience the most severe outcomes of infection. Globally, RSV is a leading cause of lower respiratory tract infections (LRTIs), contributing to an estimated 94 600 to 149 400 deaths annually, with approximately 33 million RSV-associated LRTIs resulting in 3.2 million hospital admissions. Mortality rates for RSV-associated LRTIs range from 12% to 43%, highlighting the devastating impact of this infection on young children.^1^

The burden is especially pronounced in Africa, where limited access to healthcare, malnutrition and concurrent infections such as malaria and human immunodeficiency virus (HIV) exacerbate disease outcomes.^2^ Despite its high morbidity and mortality, RSV remains under-recognised in many African countries because of inadequate surveillance and diagnostic capabilities, leading to missed opportunities for intervention and prevention.

Recent advancements in RSV prevention, particularly the development of maternal vaccines and monoclonal antibodies (mAbs), offer promising strategies to reduce the disease burden. Maternal vaccination, such as the recently approved RSV prefusion F protein vaccine (RSVpreF), enhances antibody transfer during pregnancy, providing passive immunity to infants during their most vulnerable early months. Similarly, mAbs such as palivizumab and nirsevimab directly target the virus, offering high efficacy but facing challenges related to cost and delivery. While both strategies are effective, they encounter significant barriers to implementation in low-resource settings, including affordability, infrastructure limitations and potential vaccine hesitancy.

This article critically analyses these barriers while identifying opportunities and practical solutions to optimise RSV prevention efforts in Africa. Drawing on existing evidence, the discussion emphasises actionable insights and the importance of integrating these interventions into health systems to improve outcomes for African children.

## Respiratory syncytial virus vaccines and monoclonal antibodies

Efforts to develop an effective vaccine for RSV date back to the early 1960s. Over the years, this has led to the creation of several key vaccine formulations targeting different aspects of the virus’s biology. Initially, research focused on whole-virus vaccines, which were later sidelined because of safety concerns. Subsequent developments have introduced more promising approaches, including protein sub unit vaccines and vector-based vaccines, each aiming to safely elicit a robust immune response without the risks associated with earlier formulations. Notable among recent achievements are vaccines such as the RSVpreF that target the prefusion conformation of the RSV F protein, recognised for its enhanced immunogenicity and effectiveness in preventing severe RSV infections. The RSVpreF vaccine has demonstrated up to 81.8% effectiveness in preventing medically attended severe RSV infection within the first 90 days post-birth, with sustained efficacy at 69.4% up to 180 days.^3^

Maternal RSV vaccines, such as RSVpreF, are designed to be administered during pregnancy, enhancing maternal antibody transfer to protect infants during their first months of life. The success of maternal vaccination in preventing neonatal tetanus, where targeted immunisation of pregnant women has significantly reduced newborn mortality, provides a compelling precedent for its application in RSV prevention.^4^

Similarly, the introduction of mAbs has marked a significant advancement. Particularly, the single-dose nirsevimab offers immediate, targeted protection for infants by directly neutralising the virus. This method is especially valuable for high-risk infants who may not fully benefit from maternal vaccination, such as those born prematurely or to mothers unable to access antenatal care. Nirsevimab has shown an efficacy rate of 74.5% in preventing medically attended RSV-associated LRTIs and 83.2% in preventing hospitalisations because of RSV-associated LRTIs, highlighting its potential in RSV prophylaxis.^5^

## Implementation challenges

### Economic challenges

The cost of RSV prophylactics is a significant barrier to their roll-out in Africa. Current market prices place these interventions out of reach for most health systems in low- and middle-income countries (LMICs), with RSVpreF maternal vaccines costing approximately $320.00 per dose and mAbs, such as nirsevimab, priced at $495.00 per dose. Palivizumab, at $7600.00 for a full five-dose schedule, is even more expensive and less feasible for widespread use. While evidence from modelling studies suggests that these products can be cost effective in LMICs, their affordability remains a pressing concern.^6^ The inclusion of RSV vaccines in global funding initiatives, such as Vaccine Alliance’s (GAVI) 2018 Vaccine Investment Strategy, holds promise for reducing costs and improving accessibility. However, these interventions are yet to achieve prequalification by the World Health Organization (WHO), a critical step for procurement and large-scale deployment in LMICs.

### Infrastructure and logistics

The successful delivery of RSV vaccines and mAbs relies heavily on cold chain systems to maintain their potency, requiring storage at temperatures between 2 °C and 8 °C. While most African countries have established cold chain infrastructure through their Expanded Program on Immunisation (EPI), these systems are often stretched beyond capacity. Originally designed in the late 1970s to manage a limited number of less expensive and less bulky vaccines, these supply chains have often not evolved proportionately to accommodate the increasing complexity of modern immunisation schedules.^7^

In 2016, only 19% of GAVI-supported countries in Africa met the 80% minimum threshold for the World Health Organization’s recommendations for effective vaccine management.^8^ These inefficiencies contribute to significant vaccine wastage, with some studies reporting wastage rates as high as 30%. Seasonal RSV vaccination schedules may further exacerbate this issue, particularly if vaccines are not distributed efficiently or are donated close to expiry, a practice commonly observed in Africa.

To address these challenges, integrating RSV prophylactics into existing systems will require not only logistical planning but also investments to strengthen supply chain infrastructure. A health system strengthening approach, focused on expanding cold chain capacity and improving supply chain management, is essential. Outsourcing logistics to the private sector, as demonstrated in successful pilot studies in Nigeria and South Africa, has proven to provide cost-effective solutions. Specifically, the Western Cape province of South Africa experienced significant enhancements in vaccine management and handling practices upon engaging the private sector. This outsourcing resulted in improved adherence to temperature thresholds during vaccine storage and transportation, leading to more effective vaccine management.^9^ Economically, the arrangement proved to be cost effective, with outsourcing costs aligning well with, or improving upon, government-run systems. The pilot study confirmed that private sector involvement could reduce overall delivery costs, enhance on-time delivery of vaccines and maintain critical temperature controls more efficiently than existing public sector-managed systems.

### Data and surveillance gaps

A major limitation to RSV prevention efforts in Africa is the lack of robust data on RSV incidence, seasonality and impact. National-level data on RSV hospitalisations and economic burden are critically lacking in most African countries, making it difficult to design and implement targeted interventions. A study by Li et al. revealed that RSV data in children under 5 years were only available for 8% of the population in LMICs.^10^ This scarcity in data not only hampers decision-making on vaccine deployment but also limits the ability to monitor and evaluate the effectiveness of vaccination programmes.

In 2019, the WHO launched a pilot RSV surveillance programme aimed at addressing these data gaps.^11^ This programme represents a critical first step; however, its scope remains limited, covering only select countries. To ensure that vaccination strategies are evidence-based and context-specific, this surveillance mechanism must be expanded to include all African countries. Strengthening surveillance systems can provide actionable insights into RSV seasonality and disease burden, thereby supporting more efficient vaccine deployment.

Molecular surveillance also plays a pivotal role in RSV control efforts, particularly in monitoring the emergence of escape mutants. Given the error-prone nature of RSV’s ribonucleic acid (RNA) polymerase, mutations can arise that reduce the effectiveness of mAbs or vaccines. Instances of resistance to mAbs, such as nirsevimab, have already been observed in clinical trials,^12^ underscoring the need for ongoing monitoring. Comprehensive molecular surveillance systems should aim not only to track the prevalence of known resistant mutations but also to detect new variants that may compromise current or future RSV interventions.

Additionally, the scarcity of clinical trials conducted in Africa exacerbates the lack of context-specific evidence for RSV interventions. While the efficacy and safety of vaccines like RSVpreF and mAbs such as nirsevimab have been well established in high-income countries (HICs), their performance in African settings remains poorly understood. Conducting trials in LMICs is essential to account for demographic differences, such as higher prevalence of co-morbidities like malnutrition, malaria and HIV, which could influence vaccine response. Furthermore, such studies would help identify optimal delivery strategies tailored to local healthcare systems and cultural norms.

### Vaccine hesitancy

Vaccine hesitancy remains a significant obstacle to the success of any vaccination programme. In the case of RSV, historical failures, particularly the formalin-inactivated RSV vaccine developed in the 1960s, continue to influence public perceptions. The FI-RSV vaccine, tested in children failed to prevent RSV infection and led to an unexpected severe outcome known as vaccine-enhanced respiratory disease.^13^ Children who received the vaccine experienced heightened severity of RSV illness upon subsequent natural infection, with two deaths reported during the trials. This failure left a lasting legacy of mistrust towards RSV vaccines, which persists to this day.

Contemporary concerns have been compounded by reports of adverse events associated with newer RSV vaccines in HICs. For instance, clinical trials of RSV vaccines for older adults reported rare cases of Guillain-Barré syndrome, sparking scepticism about vaccine safety.^14^

In Africa, vaccine hesitancy may be further compounded by low awareness of RSV as a significant public health threat. Studies indicate that less than 40% of surveyed populations in some regions were aware of RSV’s impact, suggesting a pressing need for targeted education campaigns.^15^ To address these challenges, culturally sensitive and community-based interventions are critical. Trusted community leaders, health workers and educators can play pivotal roles in building trust, dispelling myths and communicating the safety and efficacy of modern RSV vaccines.^16^

### Opportunities

Africa has a unique opportunity to lead the charge in RSV prophylaxis, leveraging its established maternal and child health infrastructure and innovative approaches to deliver these life-saving interventions. Maternal RSV vaccines can be integrated into established antenatal care systems, as seen with neonatal tetanus immunisation campaigns, where high coverage rates have significantly reduced newborn mortality across the continent.^17^ This infrastructure ensures that RSV prophylactics can reach infants during their most vulnerable stage, maximising their public health impact.

The continent’s clear RSV seasonality in most regions offers another critical advantage.^2,10^ Aligning vaccination schedules with seasonal peaks can optimise resource allocation, reducing vaccine wastage and ensuring timely protection. For tropical regions with less defined seasonality, phased implementation programmes guided by emerging surveillance data can refine strategies over time. Scaling the WHO pilot RSV surveillance programme across Africa will be pivotal for generating these data and tailoring interventions to local epidemiological patterns.

Advocating for WHO prequalification (PQ) represents a transformative opportunity to ensure the accessibility of RSV vaccines and mAbs. Prequalification is essential for enabling procurement by global agencies such as United Nations International Children’s Emergency Fund (UNICEF) and unlocking international funding through mechanisms like GAVI. African countries, collectively and through regional platforms like the African Medicines Agency and the African Vaccine Regulatory Forum, can play a crucial role in accelerating this process. Emphasising the disproportionate RSV burden among African children and presenting robust epidemiological data can strengthen advocacy efforts and make RSV prophylactics a global priority.

Collaborations with the private sector have proven successful in improving vaccine delivery logistics in countries like Nigeria and South Africa.^9^ Expanding such partnerships across the continent can bridge gaps in cold chain capacity and last-mile delivery, particularly in remote or underserved areas. The private sector’s expertise in logistics and financing offers a sustainable pathway to making RSV prophylactics more accessible.

Community engagement remains a cornerstone of successful vaccination campaigns. Addressing vaccine hesitancy requires culturally sensitive, community-driven approaches that build trust and raise awareness of RSV’s impact.^16,18^ Collaborating with trusted community leaders, health workers and educators can amplify the benefits of RSV prophylactics, dispel myths and foster confidence in vaccine safety. Tailored messaging, rooted in local languages and narratives, ensures that interventions resonate with diverse cultural contexts.

A complementary approach that combines maternal vaccines with mAbs offers a promising pathway to equitable RSV prevention. Maternal vaccines can provide broad, population-wide immunity through antenatal care systems. While mAbs serve as a targeted solution for high-risk infants, their limited availability in regions with poor antenatal care access remains a significant challenge.

## Conclusion

Respiratory syncytial virus poses a significant threat to child health, but recent advancements in maternal vaccines and mAbs offer promising avenues for prevention. Realising the full potential of these interventions in Africa will require addressing challenges related to affordability, infrastructure, data gaps and vaccine hesitancy. Strengthening antenatal care systems, ensuring supply chain efficiency and advocating for WHO prequalification remain critical to expanding access.

While both maternal vaccines and mAbs contribute to RSV prevention, the broader accessibility of antenatal care services across diverse settings may position maternal immunisation as a more scalable approach for population-wide protection. In contexts where mAbs remain logistically or financially constrained, maternal vaccines could represent a more practical strategy for achieving equitable coverage. By advancing these interventions within existing health systems, significant strides can be made in reducing RSV-related morbidity and mortality among children in Africa.
